# Patient and public involvement in secure mental health research: setting-specific considerations and a protocol for involvement in the CORAS study (COllaborative Risk ASsessment and management)

**DOI:** 10.1186/s40900-025-00768-2

**Published:** 2025-10-28

**Authors:** Naomi Clifford, Catherine Jeynes, Ian Callaghan, Sheena Foster, Sarah Markham, Hannah Moore, Katrina Forsyth, Brian Crosbie, Seena Fazel, Daniel Whiting

**Affiliations:** 1 Nottinghamshire Healthcare NHS Foundation Trust. D Floor, Institute of Mental Health, Jubilee Campus, Triumph Road, Nottingham, NG7 2TU UK; 2 Rethink Mental Illness, Lived Experience Programmes Team, Berkshire, UK; 3 Carer, UK; 4 King’s College London, London, UK; 5 Expert by Experience, London, UK; 6 Social Care and Society, School of Health Sciences, University of Manchester, Manchester, UK; 7 School of Medicine, University of Nottingham, Nottingham, UK; 8 Department of Psychiatry, University of Oxford, Oxford, UK; 9 Oxford Health NHS Foundation Trust, Oxford, UK

## Abstract

**Background:**

Patient and Public Involvement and Engagement (PPIE) is important in secure psychiatric research because it can help ensure that research is relevant and meaningful, and a positive experience for those participating. However, there are significant challenges to embedding PPIE in research in secure hospital settings, including practical barriers to involvement. A lack of reporting of PPIE practices makes it harder for researchers to learn from previous projects, leading to missed opportunities to improve PPIE in secure settings, and there are no current setting-specific guidelines for best practice.

The CORAS study aims to examine collaborative risk assessment within secure psychiatric settings. In this study, PPIE is fully integrated throughout the research cycle, and this protocol describes the PPIE methodology being adopted. By highlighting these approaches and principles, this protocol is intended to be used as a transferrable framework for developing best practice for PPIE in research in these settings.

**Method:**

This protocol describes the ways in which we will ensure that PPIE remains central to each stage of the research project, from the formation of a smaller grant application PPIE group, through to dissemination of outputs. We discuss principles of recruitment into the PPIE group, ensuring that all areas of the secure mental health pathway are represented, and formally embracing equality, diversity and inclusion principles through the use of an Equality Impact Assessment. We also describe the core activities of the PPIE group, including the co-design of the research materials, recruitment strategies and dissemination plans, how the impact of PPIE will be examined, and practical elements such as around reimbursement and ensuring the wellbeing of PPIE group members.

**Conclusions:**

PPIE in secure mental health service research is important and challenging. This protocol outlines how we will address these challenges and ensure that PPIE is fully embedded in the design and delivery of a large study in secure settings. Although the prospective nature of this protocol precludes the sharing of outcomes and learning from the PPIE, it can nevertheless serve as a transferrable framework for the development that is urgently required in this clinical research field, as well as allow transparent future reporting of what was achieved.

## Abstract (plain language summary)

Involving people with their own lived experience in the design and running of clinical research studies can improve the quality and impact of these studies, as well as the experience for people taking part in them. In some clinical settings this involvement might be more difficult. In secure psychiatric hospitals things like security can make it difficult for people to be involved in this way in research. There is not much guidance available for researchers on how to do this better when running studies in secure settings. The CORAS study (COllaborative Risk ASsessment and management in secure services) is a research study in these settings that is exploring how to involve patients and carers in their risk assessments and risk management plans (or safety plans). We have developed detailed plans for how to make sure that patients and carers have a central role in all areas of this study, including designing parts of the study and how best to make sure that the results of the study reach patients and carers. This paper outlines how we are planning to do this, so that other researchers designing studies in these clinical settings can understand and build on some of the practical ways of involving people with lived experience.

## Background

Secure or forensic psychiatric services typically provide intensive treatment and care for people with severe mental illnesses, learning disabilities or autism who have been in contact with the criminal justice system and/or where there have been significant risks identified in the context of mental illness [1]. These services are complex and demand significant resources, offering extended care for people with numerous acute requirements [2]. The core element of this pathway of care is the secure hospital system, which in the UK exists across three security levels, and requires around twenty percent of annual mental health funding to provide treatment for around 7,000 people [1]. In these low-, medium- or high-secure hospital settings, treatment is provided under relevant mental health legislation, including for people who have been diverted from police custody or prison at one of several potential points in the criminal justice pathway. Community forensic services also exist to provide follow-up care on discharge from such settings, in some cases under ongoing supervision frameworks overseen by the Ministry of Justice. This patient group therefore is distinct from general adult psychiatric populations and requires special consideration in terms of research involvement. In addition to close links with justice services, secure services interact with many others, including general psychiatric services, and mental health services in prison, the courts, and the community. The clinical population in secure psychiatric services is an under-represented group in healthcare research, and the complex nature of these services poses specific challenges to involving patients and carers in the design of research.

### Collaborative risk assessment and the CORAS study

Care provision within the secure pathway is predominantly driven by notions of risk, that is, the likelihood, imminence and severity of a negative event occurring [3]. Most commonly, the risk of harm to others is considered, which is explicit within service specifications [4]. However, various other risks are also taken into account, including self-harm, suicide, disengagement, relapse, or other vulnerability. Risk is a crucial factor for admission, discharge, and progressive movement through the secure pathway, guiding decisions around the potential easing of restrictions.

Risk, and associated restrictive decisions, therefore feature prominently in care provision in these settings, requiring professionals to balance both individual need and public safety. Optimising patient-centred care through the involvement of patients and their carers is a key policy goal [5]. However, making assessment of risk and aligned management decisions a collaborative process has specific challenges in secure services due to power imbalances between staff and patients [6], or where patients may have communication difficulties, are lacking insight or capacity, or are disinterested in engaging [7]. Despite this complexity, collaborative risk assessment and management has been recommended for several years [3]. Nonetheless, currently the level of collaboration in the secure pathway, and the effectiveness of approaches, including any benefits or limitations offered by specific tools, is not known [8]. This is an important evidence gap, because there is potential to improve experiences and outcomes if implemented more effectively and consistently [3, 9], and due to experiences and outcomes being likely to vary between groups, which may exacerbate inequality and poor outcomes. It may be that a more collaborative approach to risk assessment and management aligns more closely with recovery-oriented principles integrated elsewhere in mental health care [10, 11].

As a result, this topic was identified as a significant research gap and became a commissioned priority, funded by the National Institute for Health and Care Research (NIHR) Policy Research Programme (NIHR206609) [12]; namely, the present study; COllaborative Risk ASsessment and management planning across the secure mental health pathway in England (CORAS). The project aims to understand for whom, how, why, to what extent, and in what circumstances collaboration between patients, carers and professionals works. To achieve this, we will use realist methods to gather evidence on existing management of risk to provide insights on best practice interventions [13, 14]. The project will be conducted in three stages, involving:

1. A realist review to understand 1) what approaches to collaborative risk assessment and management in secure psychiatric settings have been described, and 2) how, in what circumstances and why they work for patients, carers and healthcare professionals.
2. A mixed methods study to further develop theory from the realist review: mapping current provision of collaborative risk assessment/management, barriers/facilitators to implementation, and outcomes of interest.
3. The development of a “programme theory” from the overall realist synthesis (to explain how approaches work in different scenarios) and best practice recommendations for policy and practice.

### Patient and Public Involvement in designing research in secure settings

To have meaningful impact on the care, experience and recovery of people using mental health services, it is recommended that people with lived experience are involved from the outset [15]. Patient and Public Involvement and Engagement (PPIE) in research is defined by the NIHR as research that it is undertaken ‘with’ or ‘by’ members of the public, as opposed to ‘about’, ‘to’, or ‘for’ them [16]. In health and social care research, this is thought to be important for the development and implementation of research studies that are high quality, relevant, and ethical [17, 18]. It can enhance research by ensuring focus remains on outcomes that matter to those affected and that diverse perspectives are heard [16], as well as by improving participant recruitment and experience [19].

These issues are especially important in the context of the complexity of secure settings, and so meaningfully embedding patient and public involvement into study conception and design should be considered as integral. However, there are challenges to achieving this. Practical barriers such as patients moving between different settings can disrupt engagement in projects and issues such as security, reimbursement arrangements and access to technology may also complicate participation in both PPIE and research itself, as well as the capacity of staff and services to support people’s involvement. This limits the amount of targeted research in these settings, and frequently means evidence from research in general populations is extrapolated to patients in these settings, rather than considered in a more appropriately tailored or disaggregated manner [20].

The evidence base regarding PPIE in health research generally has grown in recent years, although reporting of this element in studies is variable [21, 22]. This may be influenced by lack of universal journal policies for reporting PPIE in research, the impact of journal word limits on capacity to report PPIE information in articles [23], and PPIE being seen as an optional rather than integral part of research [21]. Underreporting of PPIE is particularly evident in secure mental health research. A recent systematic scoping review found that reporting of PPIE in research for this population is very limited, and the majority of the included papers detailed involvement of people with lived experience of prison rather than secure mental health settings [24]. Underreporting of PPIE activities in research is problematic as it limits the opportunity for researchers to learn from the experiences of others, which may lead to misused resources and counterproductive practices [16, 21].

In the CORAS study, PPIE is embedded throughout the research cycle. In this protocol we detail the plans and considerations for this involvement, and in doing so highlight transferable approaches and best practice principles for facilitating PPIE in secure mental health research. Given that the topic of the study, risk assessment, is inherently complex, a robust approach to PPIE will help maximise the potential for the study to produce meaningful findings that are clinically relevant and actionable.

## Methods

### Involvement in grant application

In exploring the extent of, and approaches to, patient and carer collaboration in risk-related decisions in the secure mental health pathway, it was a key priority to ensure that meaningful PPIE was embedded in the research design from conception. The application to undertake this project was developed in collaboration with a lived experience co-applicant (author IC, based at the charity Rethink Mental Illness). Through existing lived experience networks, a pre-submission PPIE group was formed, which included carer representation and lived experience of secure services. This involvement was funded by a pre-submission PPIE grant from the University of Nottingham. Such pre-submission grants are not universally available but have been shown to be a key facilitator for involvement at the development stage [25]. The group helped design the initial plans for how PPIE will run during the project, the equality, diversity and inclusion strategy, and key practical aspects of the proposed study design such as logistical arrangements for recruitment tailored to the secure ward setting. This ensured that in addition to meeting the objectives set by the funders, the proposed research would also focus on the issues that matter to patients and their carers. These members will continue to support the project for the duration.

### Recruitment into the PPIE group

Establishment of a PPIE group that is demographically diverse and demonstrably reflective of the adult secure pathway will be a measure of success for the study. We will recruit 10-12 participants to support the study and act in an advisory capacity. During recruitment, extra attention will be paid to diversity such as in gender, ethnicity, geographical location, and experience of secure mental health services to ensure sufficient representation. Diversity within public and patient involvement is important to ensure all groups are enabled to contribute [26], but achieving this requires resource and time [27]. Carers and relatives will be recruited to the group, and early discussions highlighted the importance of ensuring any carer experiences of feeling isolated in clinical scenarios are not mirrored by research processes that may inadvertently exclude. Inclusivity can take several forms, to ensure meaningful involvement we will strive to make research processes understandable to PPIE members, providing an appropriate level of ‘interactional expertise’ around research methods, so that individuals can use this knowledge to inform their experiential input to discussions and decisions [28]. We will actively seek membership from people with both current and former lived experience of secure services.

As the core research team includes clinicians practising in secure services, we will utilise links with the organisational networks of the secure pathway nationally to recruit people currently receiving care either within inpatient settings or in the community. Existing PPIE networks will serve as a starting point for member recruitment. It will be necessary however to widen recruitment to mitigate the risk of recruiting only those who are most accessible and possibly lacking in diversity. NHS-led regional secure care Provider Collaboratives (with whom initial PPIE members are well-linked) will be used to expand the group directly from clinical services along with involvement groups aligned to the Royal College of Psychiatrists and NHS England. Provider Collaboratives facilitate collaboration between health and social care providers and across local systems with the aim of enabling people with specialist healthcare needs (such as those requiring admission to secure mental health units) to access the right care at the right time, close to home [29].

### Core activities and co-design

As a national group, meetings will be held predominantly online, three-monthly throughout the project, to enable meaningful participation and remove potential travel or venue-based access barriers. This will also support the involvement of current patients who may not be permitted to leave their wards or who may leave only for limited amounts of time [15]. Participation will be flexible and collaboratively agreed, to be inclusive for those with specific communication needs, accessibility requirements, and personal commitments, by considering practicalities around timing, format, and communication of information. Examples of this include arranging meetings at times that suit the majority of group members and offering 1:1 meetings for those unable to attend. Group members will be consulted on whether additional training or support would optimise their contribution.

The PPIE lead will support a group member to attend and feed into the main steering group of the research project, ensuring PPIE is demonstrably central to all discussions and decisions. Following each PPIE meeting, the lead will circulate a meeting record and agreed action points for the core research team and steering group. At each PPIE meeting, actions taken following the last meeting will be fed back, so that PPIE impact is contemporaneously captured and transparent throughout the research cycle.

Core activities will include:

- Supporting with refining participant recruitment strategies and the specification of target quotas for the individual interviews.
- Co-designing the survey, interview schedules, and participant-facing documentation.
- Advising on our approach to key equality, diversity, inclusion, and health inequalities issues.
- Contributing to the interpretation of the findings in line with realist methods.
- Co-designing accessible methods and media for dissemination.

Co-design, which is bringing people together as active partners in this manner for research, is regarded as a facilitator of more insightful, meaningful research, although more evaluation is needed on the extent to which this leads to improved outcomes [30].

Additionally, if feasible, up to two PPIE members will be involved, on a voluntary basis, as peer-researchers to co-facilitate semi-structured interviews to overcome any individual participant barriers. Participants will be able to choose whether they wish to have a peer researcher from the PPIE group present. After carefully counselling peer co-facilitators to check suitability for the interviews, such as by ensuring understanding, wellbeing and readiness, we will offer, where necessary, training to cover subject knowledge, active listening skills, confidentiality and safeguarding, handling untoward circumstances, and managing distress. Peer facilitation is envisaged to take place in remote interviews, therefore not requiring peer co-facilitators to enter participants’ secure hospital environments. We will also put in place processes to support the person to manage their own wellbeing, through 1:1 meetings immediately before and after interviews and regular check-ins between interviews.

### Equality Impact Assessment

Effective PPIE involvement should be intentional, accessible and mutually beneficial, with principles of equality, diversity and inclusion embedded throughout to reduce inadvertent tokenism [18]. It is important to highlight that the clinical population for this proposed research – that is, people in contact with the adult secure mental health pathway – are, as a whole, an under-represented group in health research [31]. Considering the additional layers of inequity within this already under-served population is therefore an essential and integral part of the research. Inequalities experienced by people within mental health services include the overrepresentation of people from racially minoritised communities due to higher rates of psychiatric diagnoses and use of the Mental Health Act 1983 in this population [32], who are also subject to longer admissions to secure settings and have higher rates of readmission [33]. Additional inequalities include those with diagnoses of severe mental illness being more likely to experience additional years in poor health and having reduced life expectancies in comparison to the general population [34].

The project will be enhanced by undertaking two Equality Impact Assessments (EqIAs) to (1) support the formation and ongoing activities of an inclusive PPIE group, and (2) ensure that relevant equalities considerations are accounted for in the research as a whole. The EqIA process is an evidence-based approach to increase the relevance and quality of the research undertaken by examining how what we do can affect people positively, negatively, or neutrally, enabling researchers to amend and adapt protocols accordingly [35]. The first EqIA was completed with involvement from the PPIE group members who contributed to the funding application. This ensured that equalities factors related to all nine protected characteristics [36] were considered and an action plan devised to address potential issues hindering group participation.

### Involvement practicalities

PPIE group members will include people currently in hospital, as well as people living in the community and families and carers. Throughout we will place emphasis on making engagement with the work a positive experience [37]. Prior to recruiting any PPIE group members who are currently inpatients, initial 1:1 calls will be made to discuss wellbeing and readiness to participate. This will then be discussed with the member of the clinical team that is supporting the person to take part. As part of this contact, space will be given to ensure that clinical staff understand the nature and purpose of the patient’s participation, and the staff member’s own role in supporting this. It is not anticipated that clinical staff would require any additional training for their role, which is not to participate actively in any discussion, which is instead led by the PPIE group facilitator. Agreements will be made with the clinical team that ward staff can support the patient’s participation in PPIE meetings by arranging for ward laptops to be used in private areas of the ward. Communication from the co-ordinating team at Rethink Mental Illness therefore goes to both the group member, if they have an email address, as well as to the contact on the ward, who is asked to print out any pre-reading materials, such as agendas, previous minutes, or background reading for the meeting.

During the meetings, staff will remain present in the room, though they will not be on screen nor participate in the discussion. This is an arrangement that inpatient group members are usually used to, and does not appear to hinder their participation. Follow-up emails will be sent to group members with access to email to ask whether there was anything else they would like to say that they were unable to say during the meeting itself.

PPIE group meetings will be co-facilitated by the PPIE lead and one other experienced facilitator from Rethink Mental Illness. Meetings will begin with a group agreement to remind people of safety and the comfort of participants and for safeguarding purposes. Should anyone become distressed during a meeting, one of the co-facilitators will meet with them in a breakout room and follow up appropriately. Optional debrief meetings will be held two to three days after group meetings to allow participants to make any further contributions and to check on wellbeing.

In the highest security settings, involvement in the main PPIE group discussion is unlikely to be feasible due to security considerations precluding such patients, for example, joining an online meeting with patients in other hospital settings. Instead, a pragmatic approach will be taken whereby materials will be prepared to allow comments to be anonymously passed to the main PPIE group by linking separately with existing internal involvement groups in these settings. To achieve this, the PPIE lead will attend in person. Whilst this resource-intensive approach will not be possible for all studies, the importance of such inclusion for this study was such that this capacity is included within funding considerations. Even with this approach, only people in these settings who can access off-ward spaces within hospital to participate will be able to be involved.

### Reimbursement

All PPIE activities have been accounted for in the funding and participation is costed at a rate of £25 per hour of involvement, plus additional training, with a typical PPIE group meeting estimated to consist of two hours participation and one hour preparation. This is in line with the NIHR’s payment guidance for researchers [38]. There will be some flexibility in the use of funded time, for example at points where study resources are being co-developed.

Payment is via the Rethink Mental Illness payroll system and from a tax perspective occurs on a ‘Pay as You Earn’ (PAYE) basis, which means that tax and national insurance are deducted at source, as appropriate, avoiding participants having to complete tax self-assessments. Guidance is provided to participants who may be claiming benefits, which could possibly be impacted should payment exceed a certain amount [39]. A letter for relevant agencies (Jobcentre Plus in England) is available to explain that this is not employment and is ‘service user involvement’. Should participants decide not to accept payment in that manner, one voucher of £50 can be offered per financial year, in line with national tax rules. This is known as a ‘trivial benefit payment’ and does not accrue tax or affect benefits.

### Representation within project steering group

The steering group, composed of a variety of professionals working across the secure pathway and in aligned agencies (e.g., justice) will advise and steer the project, meeting three-monthly. Importantly, membership will include the PPIE lead and a carer representative, who will input into these meetings as a standing agenda item, ensuring that valuable lived experience insights remain at the forefront of discussions [40]. The PPIE lead will also join the core research team progress meetings to enable reciprocal sharing of updates and feedback.

### Evaluating impact

The impact of PPIE on research is underreported and inconsistently captured [41]. We will use the regular meetings with PPIE group members as a formative evaluation method to measure impact, by reflecting on the content of feedback and discussions and documenting key points, reflections and resulting actions in a PPIE impact log. The reported participation experiences of group members will also be an important aspect of ongoing evaluation, which will include feedback surveys during and at the end of the project. For a summative evaluation, these notes and surveys will be used to assess the overall process and influence of PPIE activities. We will use the Guidance for Reporting Involvement of Patients and the Public (GRIPP 2) reporting checklist to frame our reporting of involvement in a consistent, transparent and effective manner [21]. Additionally, the research team will consider and document their experiences of embedding PPIE in the research, which can provide helpful learning [42]. It is important that this sharing of experiences is transparent and includes the sharing of any negative experiences or challenges. We aim to contribute to the evidence base on PPIE in secure mental health research by reporting our experiences, providing practical advice around perceived and actual barriers, any appropriate mitigations, lessons learnt, and points of achievement.

### Ethical considerations

In a UK context, the Health Research Authority makes it clear that involvement activity does not require ethical approval, the key distinction being that the activity involves collecting opinions rather than study data. This may vary in some non-UK contexts. Although in the UK PPIE is therefore not formally governed by ethical review and approval processes in the same way as research itself, it is nevertheless essential to take an ethical and moral approach to involving members of the public and people with lived experience in research [17], especially when they may be considered a vulnerable population. To ensure our PPIE activities follow best practice, we will design all our PPIE activities according to the six standards identified by the NIHR in their current patient and public participation guidelines: communication, working together, inclusive opportunities, impact, governance and support and learning [43, 44]. One potential issue is the risk of power imbalance in such activities, mitigated here by PPIE activities being led by a member of the research team with lived experience. The issue of reimbursement for involvement (and similarly for study participation) has been debated with regards to the potential for coercion [45]. However, if undertaken proportionately such that it does not unduly influence the decision to participate, reimbursement offers a fair way to compensate participants for their time and effort and demonstrate the value of this, and in the UK is stipulated in best practice guidance [46].

## Conclusion

Public and patient involvement in research in secure mental health services is both important and specifically challenging. In this paper we have outlined the reasons for this importance, and our intention to underpin a study of collaborative risk assessment in secure mental health settings with effective PPIE at each stage. A fundamental aim of the CORAS study is that PPIE will support the design and delivery of research that is anchored in patient and carer experience and can inform recommendations for practical improvements in how collaborative risk assessment is approached. This protocol outlines how we intend to achieve that aim, and may also benefit future research by acting as a practical template for PPIE in secure psychiatric settings, for which there is currently a lack of consensus, guidance and research.

## Data Availability

No datasets were generated or analysed during the current study.
